# Endogenous expression of inactive lysine deacetylases reveals deacetylation-dependent cellular mechanisms

**DOI:** 10.1371/journal.pone.0291779

**Published:** 2023-09-18

**Authors:** Tasha B. Toro, Elena V. Skripnikova, Kiara E. Bornes, Kun Zhang, Terry J. Watt

**Affiliations:** 1 Department of Chemistry, Xavier University of Louisiana, New Orleans, LA, United States of America; 2 Division of Basic and Pharmaceutical Sciences, Xavier University of Louisiana, New Orleans, LA, United States of America; 3 Department of Computer Science, Xavier University of Louisiana, New Orleans, LA, United States of America; 4 Bioinformatics Core, Xavier University of Louisiana, New Orleans, LA, United States of America; Columbia University Irving Medical Center, UNITED STATES

## Abstract

Acetylation of lysine residues is an important and common post-translational regulatory mechanism occurring on thousands of non-histone proteins. Lysine deacetylases (KDACs or HDACs) are a family of enzymes responsible for removing acetylation. To identify the biological mechanisms regulated by individual KDACs, we created HT1080 cell lines containing chromosomal point mutations, which endogenously express either KDAC6 or KDAC8 having single inactivated catalytic domain. Engineered HT1080 cells expressing inactive KDA6 or KDAC8 domains remained viable and exhibited enhanced acetylation on known substrate proteins. RNA-seq analysis revealed that many changes in gene expression were observed when KDACs were inactivated, and that these gene sets differed significantly from knockdown and knockout cell lines. Using GO ontology, we identified several critical biological processes associated specifically with catalytic activity and others attributable to non-catalytic interactions. Treatment of wild-type cells with KDAC-specific inhibitors Tubastatin A and PCI-34051 resulted in gene expression changes distinct from those of the engineered cell lines, validating this approach as a tool for evaluating in-cell inhibitor specificity and identifying off-target effects of KDAC inhibitors. Probing the functions of specific KDAC domains using these cell lines is not equivalent to doing so using previously existing methods and provides novel insight into the catalytic functions of individual KDACs by investigating the molecular and cellular changes upon genetic inactivation.

## Introduction

Lysine acetylation has emerged as a prevalent post-translational modification (PTM) that is involved in regulation of proteins. As improved mass spectroscopy techniques have been developed, thousands of acetylated proteins have been identified from cell lysates [1–3]. Acetylation status of target proteins is mediated by acetyltransferases, which acetylate lysine residues, and deacetylases, which are responsible for removing acetyl groups, thus restoring lysine residues to their initial state. As the first deacetylases discovered were nuclear proteins which deacetylated histones, the enzyme family was initially named histone deacetylases (HDACs); however, many members of the family are found in the cytoplasm and deacetylate non-histone proteins [4–6]. To date, 18 enzymes capable of lysine deacetylation have been identified in human cells, 11 of which are metal-dependent enzymes that are known as lysine deacetylases (KDACs; also known as histone deacetylases) [7, 8]. KDACs are divided further into classes, where class I KDACs contain a single domain and include KDAC1-3 and KDAC8, while class II KDACs contain multiple domains and include KDAC4-7 and KDAC9-10. KDAC8 (class I; Uniprot ID: Q9BY41; HGNC: HDAC8) and KDAC6 (class II; Uniprot ID: Q9UBN7; HGNC: HDAC6) have been relatively well-studied compared to other KDACs. Their structures have been solved, and a handful of substrates have been identified both *in vitro* and *in vivo* [9]. While KDAC8 is composed of only a single catalytic domain, KDAC6 contains three domains, a ubiquitin-binding domain and two catalytic domains. Previous work indicates that the second catalytic domain (CD2) is mostly responsible for catalysis; however, the first catalytic domain (CD1) appears capable of deacetylating a narrower set of substrates [10, 11].

Acetylated proteins are found in all cellular compartments and on proteins that affect many critical cellular processes, implicating this PTM as a critical player in normal biology and linking misregulation of acetylation/deacetylation to many diseases. In particular, KDAC8 overexpression and misregulation has been linked to several cancers, where KDAC8 has been shown to promote tumor formation, metastasis, and drug resistance in certain situations [12]. Inhibiting KDAC8 has been shown in several experimental contexts to counteract several of these disease-promoting effects [13]. KDAC6 is thought to play a role in maintaining processes such as cell division and migration, possibly mediated by its well-characterized deacetylation of tubulin. Not surprisingly, overexpression of KDAC6, like KDAC8, has also been connected to several caners as well as other human diseases [14]. Currently, several KDAC6 specific inhibitors are being investigated in clinical and pre-clinical trials for their therapeutic potential against many diseases, including several cancers [15, 16]. However, despite these promising advances over several years and many clinical trials, no KDAC-specific inhibitors have yet been approved for use as clinical therapeutics. Only a handful of non-specific KDAC inhibitors have been approved to treat a rather small set of diseases, and these drugs tend to have off-target effects due to their lack of specificity [17].

To fully realize the therapeutic potential of targeting KDACs, we first need a greater understanding of the role of individual KDACs in cells, including identifying substrates of particular KDACs and understanding the cellular consequences of misregulation or loss-of-function of particular deacetylases. Several diseases have been linked to change in expression level of a KDAC, while others are caused by expression of a mutant form of a KDAC. Cornelia de Lange syndrome, for example, can occur as a result of one of several identified missense mutations in KDAC8, which result in expression of a mutant KDAC8 with greatly reduced activity [18]. SMC3 has been identified as a substrate of KDAC8, establishing a molecular mechanism by which loss of KDAC8 function could result in a disease state [19, 20]. Although some substrates have been directly assigned to a particular KDAC, it is not yet known which KDAC is responsible for deacetylating the majority of known acetylated proteins, and therefore very few such molecular links between KDAC activity and disease state have been established [9]. Previous work *in vitro* and *in silico* demonstrates that although the KDACs are fairly highly-conserved, they do have distinct substrate preferences [21–25]. However, we have a limited understanding of how loss of activity of a single KDAC would affect cells and contribute to disease states in humans, such as cancer. This is mostly due to the inherent technical limitations of traditional cell-based experiments. Previous work in cells has relied on either changing the expression levels of KDACs (through knockdown or overexpression) or small-molecule inhibitors. While these methods provide some useful information, they cannot accurately capitulate solely the effect of losing catalytic activity of a particular KDAC [9]. A change in KDAC expression level (i.e., knockdown or overexpression) would affect all functions of the protein, as most KDACs assemble in complexes and/or have identified roles other than catalysis [26, 27]. Furthermore, small molecule inhibitors, even ones that show some specificity between family members, are able to inhibit multiple KDACs, because of the well-conserved active site. It is unknown how much these off-target interactions will affect experimental results, leading to uncertainty in interpretation of data resulting from cell-based inhibitor studies. These caveats have limited our understanding of the roles of individual KDACs on cells.

We have addressed these issues by assessing the effects of loss of KDAC activity in genetically-engineered HT1080 cells, which are an immortalized epithelial-like cell line derived from a fibrosarcoma in a male patient [28]. HT1080 cells were especially amenable to these experiments, as they readily proliferate yet have retained a relatively normal number of chromosomes compared to other common immortalized lines [29]. Previous work has demonstrated that a single histidine to alanine point mutation in the active site of the KDACs is sufficient to reduce activity at least 5000-fold [30–32]. Using a CRISPR/Cas9 based method, we have created derivative cell lines in which the previously characterized missense mutation was introduced in the endogenous locus of either KDAC6 or KDAC8, resulting in the catalytic inactivation of the resulting KDAC while retaining expression similar to wild-type levels. Here, we present the creation of three new cell lines, HT1080 KDAC6H216A, HT1080 KDAC6H611A, and HT1080 KDAC8H143A, and an initial characterization of the cellular effects resulting from these perturbations.

## Materials and methods

### Cloning

Guide RNA (gRNA) sequences to target Cas9 to each mutation site were designed using a process preciously described by Ran et. al (Table 1) [33]. CRISPOR (crispor.tefor.net) was used to select the optimal gRNA for each target based on minimizing off-target probability and retaining proximity of the double-strand break to the targeted site [34]. Homology-directed repair templates were designed to be complementary to the 3’ end of the cleaved strand based on the work of Bialk et al (Table 1) [35]. pSpCas9(BB)-2A-Puro (PX459) V2.0 was a gift from Feng Zhang (Addgene plasmid # 62988,) [33]. To make pCas9 plasmids containing guide RNA (gRNA) to target KDACs, oligonucleotide pairs (IDT) encoding gRNA sequences were boiled in annealing buffer (10 mM Tris pH 8.0, 10 mM MgCl_2_) and allowed to slowly cool to room temperature. Annealed oligos were ligated into the BbsI site of the Cas9-encoding plasmid.

**Table 1:** Oligonucleotides used for mutant cell line creation.

gene locus	purpose	direction	sequence (5’-3’)
KDAC8H143	cloning gRNA	forward	CACCG ATAAC TGAGA AACTG ACTCA
KDAC8H143	cloning gRNA	reverse	AAACT GAGTC AGTTT CTCAG TTATC
KDAC6H216	cloning gRNA	forward	CACCG AGCCA TCCAT AAGAC TGTGC
KDAC6H216	cloning gRNA	reverse	AAACG CACAG TCTTA TGGAT GGCTC
KDAC6H611	cloning gRNA	forward	CACCG AAGCT GCATC CTGCT CTGCG
KDAC6H611	cloning gRNA	reverse	AAACC GCAGA GCAGG ATGCA GCTTC
KDAC8H143A	repair template		GAGGG GCTAC GATCA CAGCT GCCCA ATGCC TGATT GACGG AATGT GCAAA GTAGC AATTA ACTGG TCTGG AGGAT GGCAT GCCGC AAAGA AGTAA GAAAA TGACC TTTCT GTTTT CTGAC TCTTT CATTG AGTCA GTTTC TCAGT TATGT AAACT CTTAT GCTTA TTGTA TTGAC ATTTA CAGAG AGACG TGTGT GTATG
KDAC6H216A	repair template		GAGCA GGGGA CAGTC TGTCT CTGCA GAGGA GGACC CCTCA CATAC TCCAA GCTGT TATTT CCTTG TCTTG CCCAG ACCTC CTGGG CATGC TGCGC AGCAC AGTCT TATGG ATGGC TATTG CATGT TCAAC CACGT GGCTG TGGCA GCCCG CTATG CTCAA CAGAA ACAC
KDAC6H611A	repair template		GAGGG CAGGC TGTCC GATCT TAGCA CCCTG CTGCT TCCCA GGTTC TGAAT GGTGC TGCTG TGGTG CGTCC GCCTG GGCAT GCTGC AGAGC AGGAT GCAGC TTGCG GTTTT TGCTT TTTCA ACTCT GTGGC TGTGG CTGCT CGCCA TGCCC AGACT
KDAC8H143	diagnostic PCR	forward	GTCAC ACCTC AACCT ATTCC TC
KDAC8H143	diagnostic PCR	reverse	AGACT GTAAG TCCCT CGAAG A
KDAC6H216	diagnostic PCR and sequencing	forward	GTTTG AGGGC CTCCA TTACT C
KDAC6H216	diagnostic PCR and sequencing	reverse	GACTC TGTGC AAGGA GATCA A
KDAC6H611	diagnostic PCR	forward	GCAGG CTGTC CGATC TTAG
KDAC6H611	diagnostic PCR	reverse	CCAGG GAGTC CTTAC CGTAG
KDAC8H143	sequencing	forward	CCAGC CACTG AAGGG ATATT
KDAC8H143	sequencing	reverse	TCCTG ACTTT GAGCA GTGTG
KDAC6H611	sequencing	reverse	GCATG CCCAC TGATA GTCTG

### Cell growth and constructing catalytically-inactive cell lines

HT1080 cells (HT-1080 [HT1080] (ATCC CCL-121)) were obtained from and certified by ATCC. Cells were evaluated for mycoplasma status using a Venor^TM^GeM kit following the manufacturer’s instructions to ensure consistent lack of mycoplasma contamination. Cells were cultured in growth media (minimal essential medium with Earle’s balanced salts and L-glutamine (MEM) supplemented with 10% fetal bovine serum, 50 U mL^-1^ Penicillin, and 50 μg mL^-1^ Streptomycin) and incubated at 37°C with 5% CO_2_. HT1080 cells were genetically-modified using a modified version of a previously published CRISPR protocol [33]. Lipofectamine 2000 (Invitrogen) was diluted 1:25 in MEM media and incubated with 1.0 μg pSpCas9(BB)-2A-Puro (PX459) V2.0 containing locus-specific gRNA to introduce KDAC6H216A, KDAC6H611A, or KDAC8H143A. The mixture was added to HT1080 cells at 80–90% confluence in a 12-well plate. After 2 hr incubation at 37°C with 5% CO_2_, 40 pmol of ssDNA repair template incubated with Lipofectamine 2000 as described above was added to the cells. 24 hr after transfection, 2.0 μg mL^-1^ puromycin was added to cells.

After an additional 72 hr incubation, transfected cells were trypsinized, serially diluted in growth media and plated in 96-well plates to achieve single colony dilutions [33]. Cells were incubated for 7 days at 37°C with 5% CO_2_ and then scored to identify wells containing a single colony. Single colonies were incubated until they reached a density of at least several hundred cells, then passaged into a 24-well plate. Once confluent, cells were passaged again and harvested for genomic DNA isolation, which was performed using the quick DNA microprep kit (Zymo Research). Candidates were genotyped first by PCR and restriction digest, using a silent SphI restriction site introduced in the repair templates. All PCR reactions were performed using an initial 95°C step for 5 minutes, followed by 40 cycles of 95°C for 30 seconds, 62°C for 30 seconds, 72°C for 1 minute, then a final elongation step at 72°C for 10 minutes. PCR products from clones that showed the expected digestion pattern were subjected to DNA sequencing to confirm the presence of only the intended mutations. Wild-type HT1080 cells were also sequenced using the same oligonucleotides as controls. Oligonucleotide sequences used for diagnostic PCR and sequencing are listed in Table 1. For HT1080 KDAC6H216A, three independent cell lines were obtained and pooled for all subsequent experiments.

### Immunoblots

For protein-level analysis of KDAC expression and protein acetylation, WT and mutant cell lines were grown under standard conditions and approximately 3x10^6^ cells were harvested using standard trypsinization methods and centrifugation at 125 xg for 7 minutes, then cell pellets were stored at -80°C. Pellets were thawed in lysis buffer (30 mM MOPS pH 8.0, 150 mM KCl, 5% glycerol, 10 mM EDTA, 2 μM trichostatin A, 10 mM nicotinamide, 1X HALT protease inhibitor [Thermo Scientific]) on ice for 5 minutes, vortexed for 30 s, and sonicated at 50% max power for 1 s. 50 μg of lysate was loaded into a gel, then transferred to a nitrocellulose membrane in transfer buffer (25 mM Tris pH 7.5, 190 mM glycine, 20% ethanol) at 50V for 1 hour. Blots were blocked with 5% non-fat dry milk in 1X TBS-T (20 mM Tris pH 7.5, 150 mM NaCl, 0.1% Tween-20) for 30 min, then probed with antibodies diluted 1:1000 in 5% non-fat dry milk in 1X TBS-T (approximately 3–5 μg total antibody) overnight at 4°C with rocking. The following antibodies were used to probe for each protein: β-Actin (Developmental Hybridoma Studies Bank JLA20), KDAC6 (Santa Cruz Biotechnology sc-28386), KDAC8 (Cell Signaling Technologies 66042), SMC3 (Cell Signaling Technologies 5696), SMC3-acetyl-K105/K106 (Sigma Aldrich MABE1073), β-Tubulin (Developmental Hybridoma Studies Bank E7), α-Tubulin-acetyl-K40 (Cell Signaling Technologies 5335), Histone3 (Cell Signaling Technologies 4499), Histone H3-acetyl-K9 (Cell Signaling Technologies 9649), Histone H3-acetyl-K18 (Cell Signaling Technologies 13998), Histone H3-acetyl-K27 (Cell Signaling Technologies 8173). Subsequently, a 1:5000 dilution of appropriate secondary antibody (Invitrogen 31464 or 31432) in 5% non-fat dry milk in 1X TBS-T was incubated on the blot at room temperature for one hour and then detected using either Supersignal West Pico Plus Chemiluminescent Substrate (ThermoFisher) or Supersignal West Femto Maximum Sensitivity Substrate (ThermoFisher) according to the manufacturer’s instructions. Imaging was performed on a LI-COR Odyssey Fc imager. Immunoblot images were processed uniformly with a linear intensity range and for the maximum intensity range without exceeding saturation in any bands, except in long exposures in which the intensity range of the second most intense band was maximized while overexposing the most intense band.

### Gene expression analysis

For real-time quantitative PCR (qPCR) analysis of KDAC genes, WT and mutant cell lines were grown under standard conditions and approximately 3x10^6^ cells were harvested using standard trypsinization methods and centrifugation at 125 xg for 7 minutes, then cell pellets were stored at -80°C. High quality RNA was extracted from two biological replicates for each cell line using RNeasy Plus mini kits (Qiagen). RNA concentration and chemical purity was assessed spectroscopically using a Nanodrop spectrophotometer (Thermo Fisher), and lack of degradation was established using an Experion automated electrophoresis system (Bio-rad). cDNA was synthesized from 4.75 μg of RNA using RT2 First Strand kit (Qiagen). Reactions were assembled containing cDNA, gene specific primers (Table 2), [36–38] and RT^2^ SYBR green Fluor qPCR mastermix (Qiagen) and reacted in an iCycler with myiQ real-time PCR detection system (Bio-Rad). PUM1 and PGK1 were used as referenced genes [37, 38]. The amplification protocol was 95°C for 5 min, then 45 cycles of 95°C for 10 s, 60°C for 30 s. For each gene tested, ≥ 2 technical replicates were collected and averaged, then the ΔC_T_ values for the two controls averaged for each sample. Fold change expression was calculated using the 2^-ΔΔCT^ method for each sample against wild-type cells at three cDNA concentrations [39]. The final fold change for each sample is the average of the 2^-ΔΔCT^ from the three cDNA concentrations, each from two biological replicates (i.e., at least six distinct replicates). All of the resulting fold change values for a particular cell line and KDAC (minimum of 6) were averaged and the standard deviation calculated. KDACs with expression fold change significantly different from one were calculated using a two-tailed, one-sample t-test using a p value threshold of 0.01.

**Table 2:** Oligonucleotides used for qPCR.

gene	direction	sequence	reference
KDAC8	forward	CGGCC AGACC GCAAT G	[36]
KDAC8	reverse	CACAT GCTTC AGATT CCCTT T	[36]
KDAC6	forward	TGCCT CTGGG ATGAC AGCTT	[36]
KDAC6	reverse	CCTGG ATCAG TTGCT CCTTG A	[36]
PUM1	forward	AGTGG GGGAC TAGGC GTTAG	[37]
PUM1	reverse	GTTTT CATCA CTGTC TGCAT CC	[37]
PGK1	forward	GGAGA ACCTC CGCTT TCAT	[38]
PGK1	reverse	GCTGG CTCGG CTTTA ACC	[38]

For RNA-seq, cells were grown as described above and approximately 3x10^6^ cells were trypsinized, centrifuged at 135 xg for 7 minutes, stored in media containing 5% glycerol, and shipped on dry ice to CD Genomics for processing. For inhibitor treated cells, 1.0 μM Tubastatin A (Cayman Chemical) or 5.0 μM PCI-34051 (Cayman Chemical) were added to cell cultures at 50% confluency. 48 hrs after treatment, cells were harvested and prepared for shipment using the same method described above for untreated cells. A total of four biological replicates were analyzed for wild-type HT1080 cells and each mutant cell line, and three for inhibitor-treated cells. A minimum of 3.5 μg total RNA was extracted from each replicate and integrity validated, followed by rRNA depletion (Ribo-Zero, Illumina). cDNA was synthesized with adapters (Illumina PE150) for pair-end reads, and a minimum of 4x10^7^ reads per sample performed on the Illumina NovaSeq 6000 platform. The mRNA data is available as GSE228549. Preprocessing of the resulting data and removal of the adapter sequences was performed with Trimmomatic (www.usadellab.org/cms/index.php?page=trimmomatic), using the appropriate settings for the adapters, leading and trailing quality minimum of 3, a sliding quality window of 4:15, and a minimum read length of 36 [40]. Reads were aligned to the human genome primary assembly (GRCh38), annotated, and counted using bowtie 2 (bowtie-bio.sourceforge.net/bowtie2/index.shtml) and RSEM (deweylab.biostat.wisc.edu/rsem/) using default parameters [41, 42]. Differential expression of cell lines and inhibitor-treated cells versus wild-type was processed using TXImport (github.com/mikelove/tximport) and evaluated using DESeq2 (bioconductor.org/packages/release/bioc/html/DESeq2.html) [43, 44]. Statistically significant changes were determined using an initial p value of 0.01 followed by false discovery rate adjustment with α = 0.01 [45]. For existing RNA-seq data sets, differential expression and statistical analysis of the knockout line versus the corresponding wild-type was performed by the same method using the annotated counts provided by the authors. For existing microarray data sets, differential expression of the knockdown line versus the corresponding wild-type was determined using a detection p value < 0.001 for both samples and a fold change of at least ±1.25 using the annotated intensities provided by the authors.

### Gene ontology (GO) analysis

For gene ontology (GO) analysis, the basic ontology set 2022-10-07 and GO human annotation set 2022-09-17 were utilized. The reference set was all possible human genes as annotated for RNA-seq analysis. Statistically significant up-regulated and down-regulated genes compared to untreated wild-type cells were analyzed independently for each cell line and treatment. A one-tailed Fisher Exact test for overrepresentation of genes within each GO term under the *biological_process* group was calculated, using an initial significance threshold of p = 0.01 in SciPy 1.8 [46]. Only *is_a* and *part_of* GO relationships were utilized. For identifying all potentially significant GO terms, we used a false discovery rate adjustment with α = 0.01 [45]. To more stringently identify the most specific GO terms associated with the expression changes and eliminate the dependency effects within the GO framework, we utilized the elim method with p = 0.01 with the Bonferroni correction [47]. GO graphs were prepared using Graphviz to visualize the relationships between GO terms [48].

## Results

### Cell lines endogenously expressing catalytically inactive KDACs demonstrate enhanced acetylation on substrates

**Fig 1:**
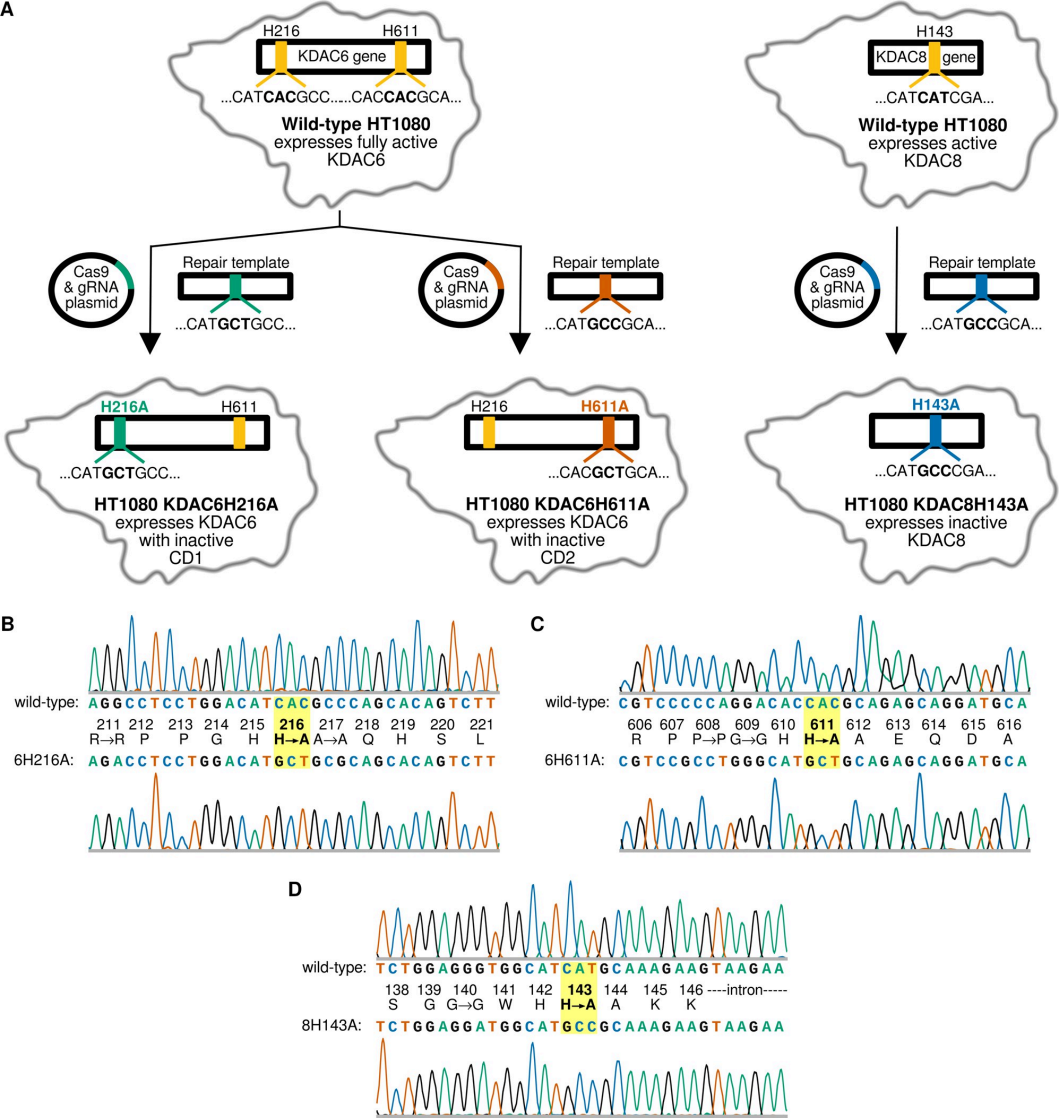
Creation of HT1080 cell lines with catalytically inactivated KDAC6 and KDAC8. (A) Schematic representation of HT1080 KDAC6H216A (green), HT1080 KDAC6H611A (red), and HT1080 KDAC8H143A (blue) cell line creation. Genes subject to mutagenesis are shown in a cartoon representation of the HT1080 cells. Wild-type codons are shown in yellow. In each resulting cell line, a single point mutation inactivated a specific catalytic domain. (B) Chromatogram from HT1080 wild-type cells (top) and HT1080 KDAC6H216A cells (bottom) surrounding the mutation. The corresponding amino acid residue positions and the resulting protein sequence are annotated in the center. (C) Same as B except for the locus corresponding to the HT1080 KDAC6H611A cells. (D) Same as B except for the locus corresponding to the HT1080 KDAC8H143A cells.

Using the CRISPR/Cas9 system, we created genetically engineered HT1080 cell lines, where each contains a single inactivated catalytic domain in either the endogenous KDAC6 or KDAC8 gene while retaining gene expression (Fig 1A). Because KDAC6 contains two catalytic domains, each of which is capable of catalysis in at least some instances, we mutated each domain independently [10, 11]. Attempts to construct a cell line with mutations in both catalytic domains of KDAC6 were unsuccessful, likely because the cells containing both mutations were not viable. In all cases, we mutated a conserved histidine in the active site, which has previously been reported to be required for catalysis [30, 32]. In total, this resulted in three mutant cell lines: HT1080 KDAC6H216A, HT1080 KDAC6H611A, and HT1080 KDAC8H143A. As HT1080 cells were derived from a male patient and both KDAC6 and KDAC8 genes reside on the X chromosome, only one copy of the gene needed to be changed in each instance. The presence of the intended mutations was confirmed by sequencing for each cell line (Fig 1B–1D). In addition to the missense mutation resulting in the histidine to alanine change in each cell line, we also introduced non-coding mutations to facilitate diagnostic screening. Furthermore, during the creation of the HT1080 KDAC8H143A cell line, a single base (G) was inadvertently inserted 47 bases after the intended mutation site, which is 40 bases into an intron and has no apparent effect.

**Fig 2:**
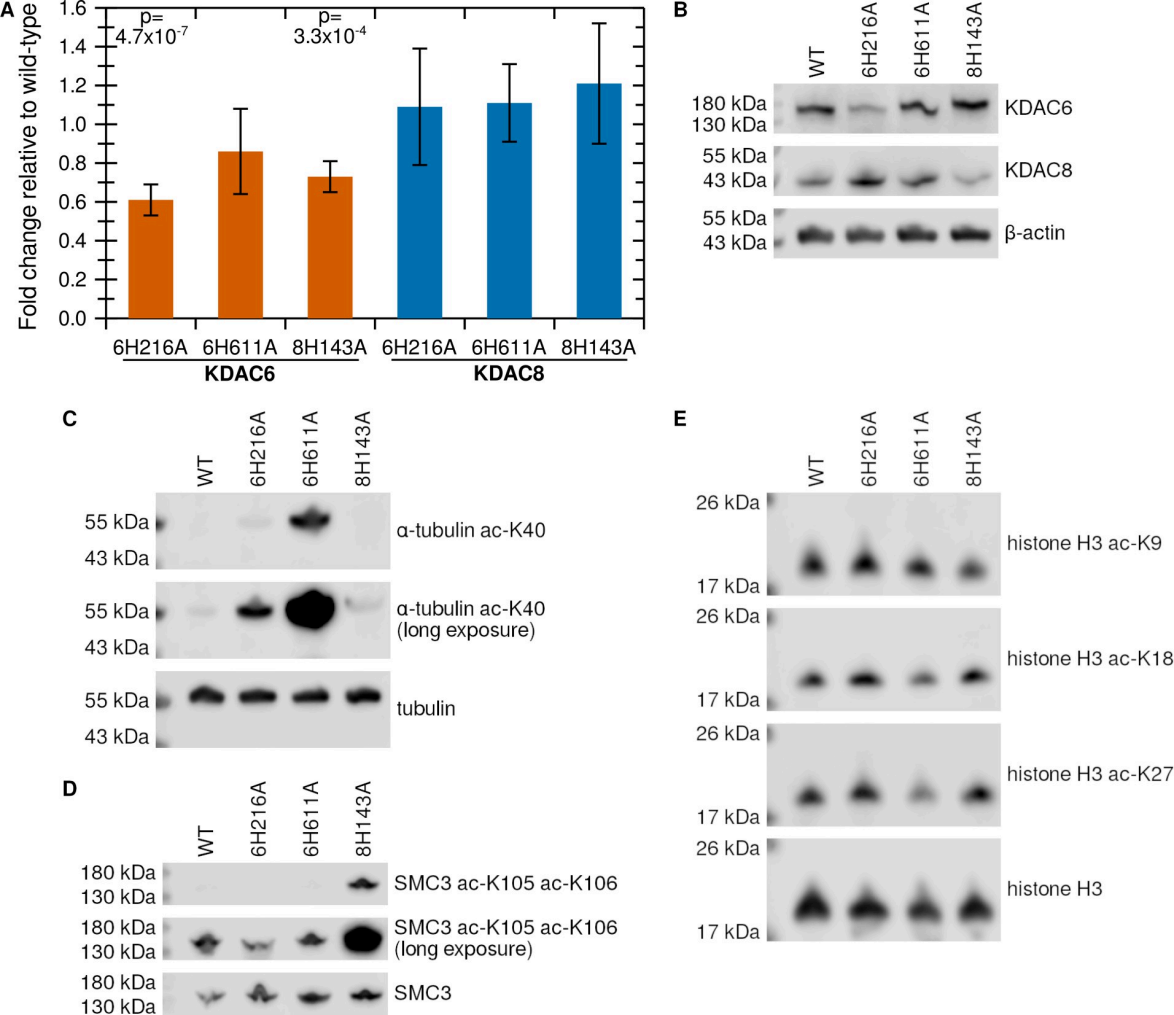
HT1080 cell lines express endogenous levels of inactive KDACs. (A) qPCR was used to measure compare expression levels of KDAC6 (red) and KDAC8 (blue) in mutant cell lines versus wild-type cells. Each bar represents the relative fold-change calculated using the ΔΔC_T_ method, and error bars represent the standard deviation of at least six replicates. P values are shown for cell lines that demonstrate a significant difference in expression levels compared to wild-type. (B) Immunoblots for KDAC6 and KDAC8 in 50 μg of lysate from each cell line. Actin was probed in 5 μg of the same lysate from each cell line as a loading control. (C) Immunoblots for tubulin and acetylated tubulin in 50 μg of lysate from each cell line. The center panel is an over-exposed version of the top panel to allow comparison of weaker signals. KDAC6 mutant cell lines exhibit increased tubulin acetylation (decreased deacetylation). (D) Immunoblots for SMC3 and acetylated SMC3 in 50 μg of lysate from each cell line. The center panel is an over-exposed version of the top panel to allow comparison of weaker signals. HT1080 KDAC8H143A exhibits increased SMC3 acetylation (decreased deacetylation). (E) Immunoblots for histone H3 and acetylated histone H3 in 50 μg of lysate from each cell line. There is no apparent significant increase in acetylation of histone H3 in the mutant cell lines.

We performed qPCR on each of the cell lines and compared the expression of KDAC6 and KDAC8 to expression in wild-type HT1080 cells to ensure that the mutant cell lines were still expressing KDAC6 and KDAC8. The expression of KDAC8 was equivalent to expression in wild-type HT1080 cells in all mutant cell lines, while KDAC6 expression was slightly lower in the HT1080 KDAC6H216A and HT1080 KDAC8H143A cell lines. Interestingly, expression of KDAC6H611A was equivalent to wild-type expression (Fig 2A). Immunoblot analysis reveals that mutant proteins were present in their respective cell lines. The relative levels of KDAC6H216A and KDAC6H611A compared to the wild-type line agree well with the expression data, while the protein level of KDAC8H143A appears slightly lower than in the wild-type line while remaining easily detectable (Fig 2B). To validate that the expressed proteins are inactivated, we measured acetylation of known substrate proteins. As expected, the acetylation of α-tubulin at K40 is enhanced when KDAC6 CD2 is inactivated (Fig 2C), indicating a lack of deacetylation by KDAC6H611A. We also observed a lesser enhancement of tubulin acetylation in the HT1080 KDAC6H216A cell line, possibly due to the slightly reduced expression of the KDAC6H216A mutant protein which could indirectly result in reduced deacetylation. Similarly, acetylation of SMC3 at K105/K106 is enhanced when KDAC8 is inactivated (Fig 2D), indicating a lack of deacetylation by KDAC8H143A. In contrast, histone H3 acetylation is not enhanced in any of the mutant cell lines compared to wild-type (Fig 2E). Overall, these data indicated that phenotypic changes in the mutant cell lines can be specifically attributed to a targeted loss of catalysis, and not to loss of KDAC expression or to global, non-specific changes in protein acetylation. Thus, these cell lines provide a new method for studying the effects of deacetylation, as the inactivation is perfectly specific to one catalytic site and non-catalytic functions of the inactivated KDAC should be retained.

### Catalytic inactivation of KDAC6 or KDAC8 lead to distinct effects on gene expression

**Fig 3:**
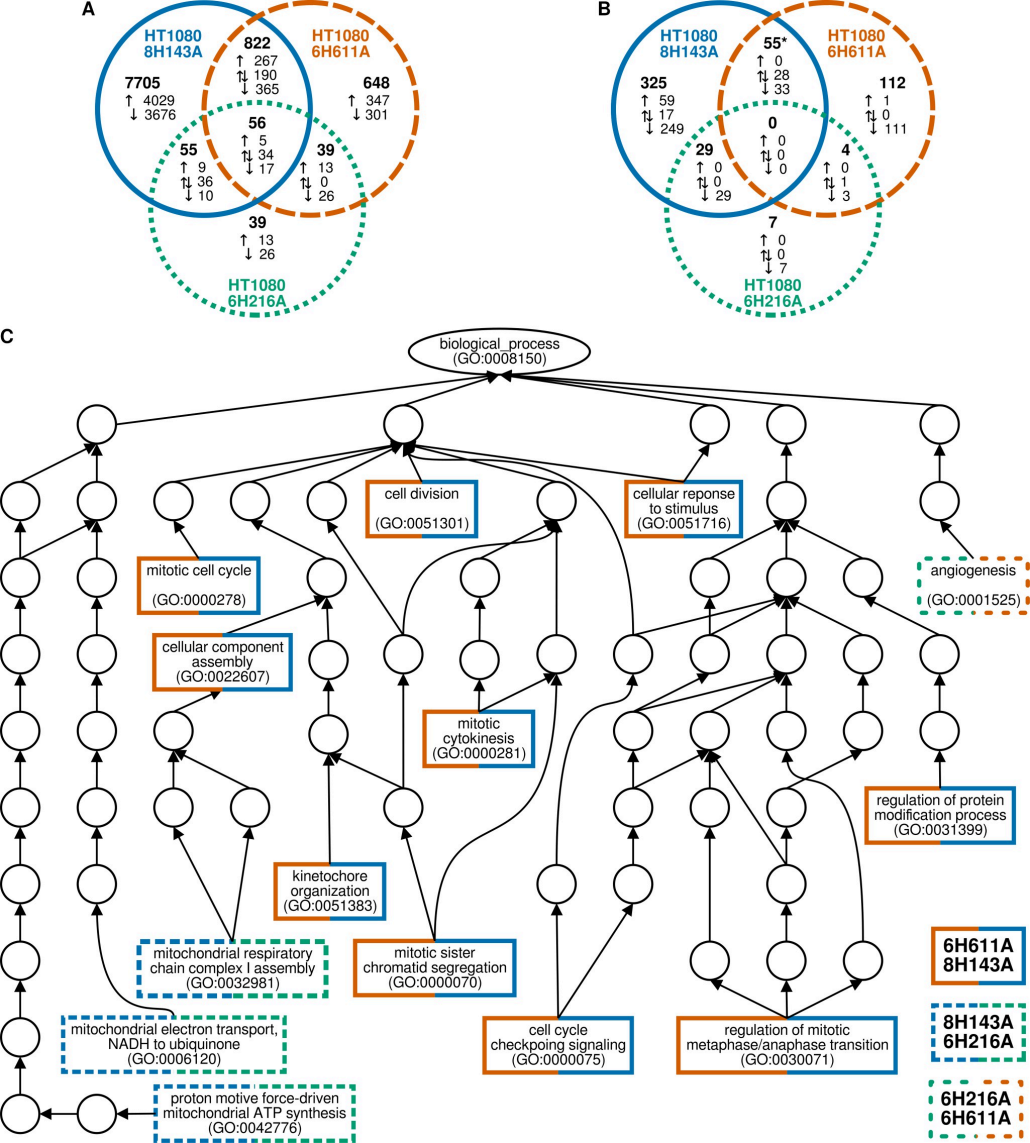
Effect of KDAC6 and KDAC8 catalytic inactivation on gene expression. (A) Venn diagram representing the number of genes for which expression level was significantly changed compared to HT1080 WT cells. Total genes in each group are shown in bold. Genes are broken down into upregulated (↑) or dowregulated (↓) for each cell line. Overlapping genes which changed expression in two or more cell lines are indicated as upregulated in all cell lines, downregulated in all cell lines, or a mixture (⇅). (B) Venn diagram representing the number of GO terms associated with genes with expression level changes compared to wild-type for each cell line (bold). GO terms associated with upregulated genes (↑), downregulated genes (↓), and both (⇅) are noted. Genes associated with the GO terms are overrepresented in the sets of expression changes based on an adjusted p value using the false discovery rate method. * denotes totals where some GO terms are associated with expression changes in multiple directions in overlap sets, resulting in a total that is less than the sum of the parts. (C) GO terms representing biological processes that are changed in multiple cell lines are shown in a hierarchical graph of GO terms. For each cell line pairwise comparison, in the event that multiple GO terms are associated, the most specific (lowest hierarchy) GO term in common is displayed. The graph was constructed using the shortest possible path to link each shared GO term to the *biological_process* ontology. Circles represent GO terms that link terms associated with catalytic inactivation to the top level of *biological_process*.

After establishing the mutant cell lines, we performed RNA-seq experiments to compare the transcriptomes of the mutant cell lines to wild-type cells. While this experiment could not identify direct substrates of KDAC6 and KDAC8, it did reveal the cellular consequences of losing deacetylation activity of each of these enzymes. We identified transcripts that were significantly increased or decreased in each cell line compared to wild-type to identify genes that were upregulated and downregulated in each cell line (S2–S4 Tables). Consistent with the qPCR data, expression of KDAC6 and KDAC8 did not significantly change in any of the cell lines. However, KDAC9 was downregulated in HT1080 KDAC6H611A while expression levels of several deacetylases, both KDACs and sirtuins, were significantly perturbed in the HT1080 KDAC8H143A cell line (S2–S4 Tables). Overwhelmingly, the most changes in expression were observed when KDAC8 was inactivated (Fig 3A). There were approximately 5.5-fold more gene expression changes identified when KDAC8 was inactivated (HT1080 KDAC8H143A), compared to inactivation of the second catalytic domain (CD2) of KDAC6 (HT1080 KDAC6H611A). Inactivating the first catalytic domain of KDAC6 (CD1; HT1080 KDAC6H216A) resulted in an order of magnitude less gene changes than inactivation of KDAC6 CD2. Furthermore, there was not a high degree of overlap in genes affected by inactivation of the two KDAC6 catalytic domains. Perhaps more surprising was the higher number of genes affected both by inactivation of KDAC8 and KDAC6.

Next, we wanted to understand which cellular processes were being affected by the loss of deacetylation activity for each cell line. To do this, we used the set of genes that were up or down regulated in each cell line to identify biological processes in the GO database in which the affected genes were overrepresented (S5 Table and S1–S5 Figs). We identified a large number of GO terms involved in a range of cellular processes associated with the HT1080 KDAC8H143A and HT1080 KDAC6H611A cell lines. However, there were only a handful of GO terms associated with HT1080 KDAC6H216A and all of the identified terms were associated with the downregulated gene set. Due in part to the large numbers of genes affected by perturbing KDAC8, several GO terms were associated with both the up and downregulated gene sets in the HT1080 KDAC8H143A cell line. As with the genes, there was only modest overlap between the two KDAC6 mutant lines (Fig 3B). Notably, there were no GO terms associated with all three cell lines. We then used a more stringent statistical method that identified the most specific GO terms associated with the gene changes for each cell line (S6 Table), from which we generated a non-redundant list of GO terms associated with each mutation that excludes all terms for which another significant term is a hierarchical descendant (Table 3). Overall, these results indicated that the catalytic domains we investigated each had individual functions in the cells.

**Table 3:** Most specific biological processes affected by KDAC inactivation.

Cell line	GO term	GO ID	p value*	direction
6H216A	proton motive force-driven mitochondrial ATP synthesis	GO:0042776	1.0x10^-4^	down
6H216A	mitochondrial electron transport, NADH to ubiquinone	GO:0006120	3.0x10^-4^	down
6H216A	angiogenesis	GO:0001525	7.1x10^-4^	down
6H611A	cell-cell adhesion	GO:0098609	1.8x10^-5^	up
6H611A	cell division	GO:0051301	5.1x10^-7^	down
6H611A	regulation of cell migration	GO:0030334	9.6x10^-6^	down
6H611A	regulation of cell shape	GO:0008360	2.8x10^-5^	down
6H611A	cell migration	GO:0016477	3.1x10^-5^	down
6H611A	anatomical structure formation involved in morphogenesis	GO:0048646	1.5x10^-4^	down
6H611A	positive regulation of cell-cell adhesion	GO:0022409	1.6x10^-4^	down
6H611A	chromosome segregation	GO:0007059	2.0x10^-4^	down
6H611A	regulation of intracellular signal transduction	GO:1902531	3.4x10^-4^	down
6H611A	negative regulation of signal transduction	GO:0009968	5.3x10^-4^	down
6H611A	cytoskeleton organization	GO:0007010	1.0x10^-3^	down
6H611A	mitotic spindle assembly checkpoint signaling	GO:0007094	1.2x10^-3^	down
6H611A	regulation of catalytic activity	GO:0050790	2.1x10^-3^	down
6H611A	nucleosome assembly	GO:0006334	2.5x10^-3^	down
6H611A	positive regulation of developmental process	GO:0051094	3.7x10^-3^	down
6H611A	regulation of cell population proliferation	GO:0042127	4.9x10^-3^	down
6H611A	neuron projection morphogenesis	GO:0048812	8.4x10^-3^	down
6H611A	mitotic cell cycle	GO:0000278	9.2x10^-3^	down
6H611A	regulation of mitotic nuclear division	GO:0007088	9.5x10^-3^	down
8H143A	intracellular signal transduction	GO:0035556	1.6x10^-7^	up
8H143A	positive regulation of DNA-templated transcription	GO:0045893	4.4x10^-5^	up
8H143A	negative regulation of transcription by RNA polymerase II	GO:0000122	5.4x10^-5^	up
8H143A	protein modification process	GO:0036211	5.4x10^-4^	up
8H143A	phosphorylation	GO:0016310	7.1x10^-4^	up
8H143A	cellular response to DNA damage stimulus	GO:0006974	1.4x10^-3^	up
8H143A	nucleic acid metabolic process	GO:0090304	2.1x10^-3^	up
8H143A	positive regulation of axonogenesis	GO:0050772	3.5x10^-3^	up
8H143A	chromatin organization	GO:0006325	3.7x10^-3^	up
8H143A	regulation of signal transduction	GO:0009966	6.7x10^-3^	up
8H143A	cytoplasmic translation	GO:0002181	4.3x10^-26^	down
8H143A	mitochondrial translation	GO:0032543	2.7x10^-21^	down
8H143A	establishment of localization in cell	GO:0051649	8.3x10^-16^	down
8H143A	cell division	GO:0051301	1.5x10^-11^	down
8H143A	proton motive force-driven mitochondrial ATP synthesis	GO:0042776	3.4x10^-11^	down
8H143A	proteasome-mediated ubiquitin-dependent protein catabolic process	GO:0043161	1.5x10^-9^	down
8H143A	mitochondrial respiratory chain complex I assembly	GO:0032981	3.9x10^-8^	down
8H143A	mitochondrial electron transport, NADH to ubiquinone	GO:0006120	2.2x10^-7^	down
8H143A	rRNA processing	GO:0006364	6.0x10^-7^	down
8H143A	protein folding	GO:0006457	1.4x10^-6^	down
8H143A	DNA repair	GO:0006281	1.9x10^-5^	down
8H143A	regulation of translation	GO:0006417	2.4x10^-5^	down
8H143A	cell cycle	GO:0007049	3.0x10^-5^	down
8H143A	endoplasmic reticulum to Golgi vesicle-mediated transport	GO:0006888	5.7x10^-5^	down
8H143A	protein N-linked glycosylation via asparagine	GO:0018279	7.6x10^-5^	down
8H143A	ribonucleoprotein complex assembly	GO:0022618	4.1x10^-4^	down
8H143A	protein targeting	GO:0006605	6.5x10^-4^	down
8H143A	regulation of macroautophagy	GO:0016241	7.1x10^-4^	down
8H143A	regulation of protein ubiquitination	GO:0031396	1.6x10^-3^	down
8H143A	protein insertion into mitochondrial outer membrane	GO:0045040	1.7x10^-3^	down
8H143A	tRNA metabolic process	GO:0006399	2.3x10^-3^	down
8H143A	cholesterol biosynthetic process	GO:0006695	2.3x10^-3^	down
8H143A	establishment of protein localization to organelle	GO:0072594	2.6x10^-3^	down
8H143A	protein stabilization	GO:0050821	3.0x10^-3^	down
8H143A	nucleotide-sugar metabolic process	GO:0009225	3.7x10^-3^	down
8H143A	protein localization to mitochondrion	GO:0070585	3.9x10^-3^	down
8H143A	DNA duplex unwinding	GO:0032508	4.2x10^-3^	down
8H143A	DNA recombination	GO:0006310	4.5x10^-3^	down
8H143A	regulation of DNA metabolic process	GO:0051052	4.9x10^-3^	down
8H143A	regulation of proteasomal protein catabolic process	GO:0061136	5.2x10^-3^	down
8H143A	regulation of mitotic metaphase/anaphase transition	GO:0030071	5.3x10^-3^	down
8H143A	regulation of response to DNA damage stimulus	GO:2001020	8.0x10^-3^	down
8H143A	microtubule cytoskeleton organization involved in mitosis	GO:1902850	9.6x10^-3^	down

* Adjusted for Bonferroni correction as described in the methods, with significance threshold of p = 1.0x10^-2^.

To determine what processes might be shared between KDAC6 and KDAC8, we compared the overlapping GO terms for each pair of cell lines. A GO graph shows the processes affected in more than one cell line (Fig 3C). For simplicity, we are only displaying the most specific term when several terms were shared from a single branch. The only process associated with both CD1 and CD2 of KDAC6 was angiogenesis. KDAC8 and KDAC6 CD1 were both involved in processes related to ATP synthesis, while KDAC8 and KDAC6 CD2 mostly shared processes related to the cell cycle.

### Catalytic inactivation is not equivalent to knockout or inhibition

**Fig 4:**
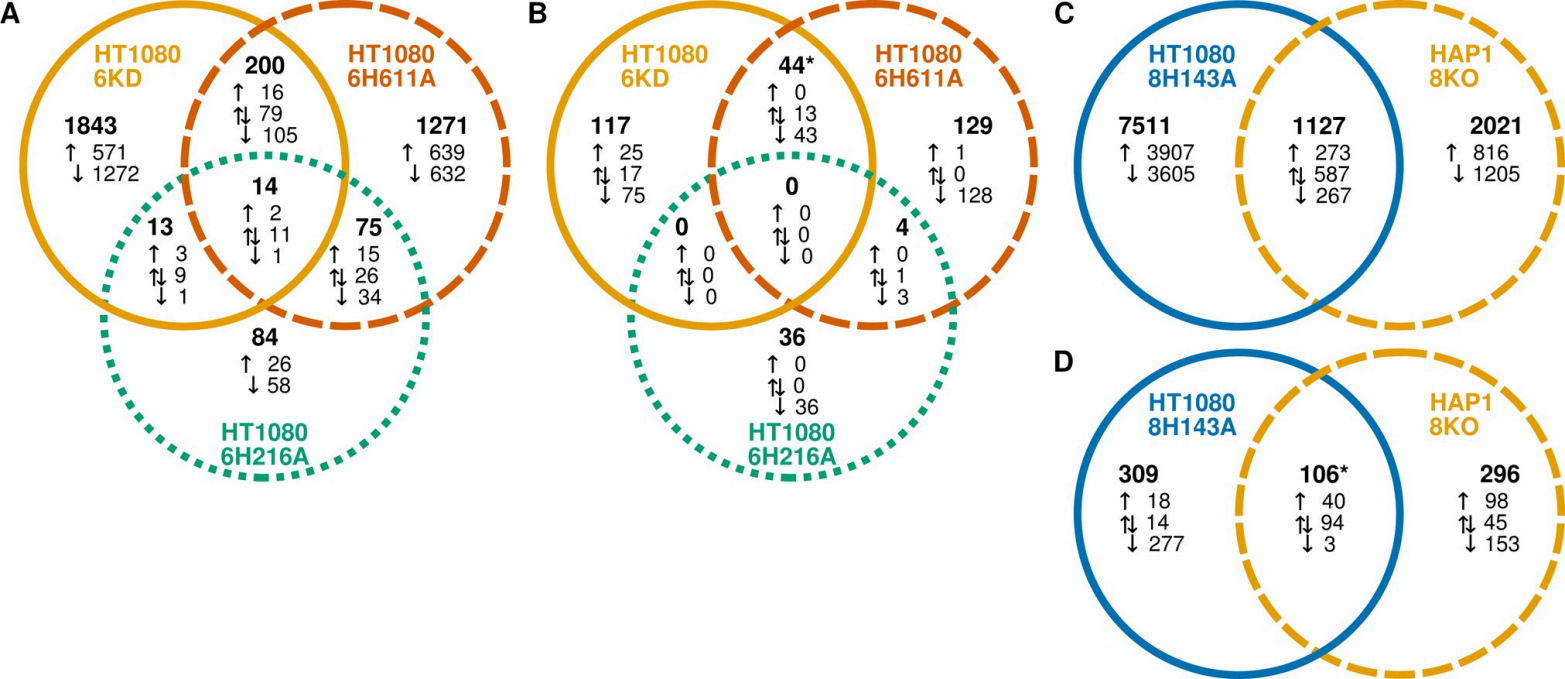
Effect of KDAC6 and KDAC8 catalytic inactivation on gene expression is not equivalent to loss of the KDAC. (A) Venn diagram comparing the number of genes with significant changes in expression level for each cell line perturbing KDAC6 compared to wild-type HT1080 cells. (B) Venn diagram comparing the GO terms associated with gene changes for each KDAC6 mutant line compared to wild-type. (C) Venn diagram comparing the number of genes with significant changes in expression level for HT1080 KDAC8H143A vs HT1080 WT and HAP1 KDAC8KO vs HAP1 WT cells. (D) Venn diagram comparing the GO terms associated with gene changes for HT1080 KDAC8H143A vs HT1080 WT and HAP1 KDAC8KO vs HAP1 WT cells. Gene counts and GO terms are represented in the same way as for Fig 3.

Prior to this work, the effects of KDACs on cells have often been investigated using knockout or knockdown (KD) cell lines. We hypothesized that loss of a KDAC protein would not have the same cellular effect as catalytic inactivation of the endogenous KDAC. A previously published report included gene expression analysis by microarray of KDAC6 knockdown in HT1080 cells [49]. We analyzed data from this experiment in the same manner as our RNA-seq data to construct sets of genes that were up and downregulated in response to KDAC6 knockdown (S7 Table). Consistent with our hypothesis, there was very little overlap between expression changes in the KDAC6 knockdown experiment compared to either the HT1080 KDAC6H216A or the HT1080 KDAC6H611A cell line (Fig 4A). Despite the number of changed genes in the KDAC6 knockdown experiment, there were very few GO terms associated with the gene changes (S5 and S6 Tables). Of these, the only GO terms in common with the catalytically inactivated cells were anatomical structure development (KDAC6 KD and KDAC6H216A) and developmental process (KDAC6 KD, KDAC6H216A, and KDAC6H611A; Fig 4B). In both cases, these processes were upregulated when KDAC6 was knocked down, but downregulated when KDAC6 was catalytically inactivated. These data clearly demonstrate that loss of KDAC6 catalytic activity is functionally distinct from loss of KDAC6 expression. Thus, the effects on gene expression of losing KDAC6 entirely are dramatically different from losing KDAC6 catalytic activity while maintaining expression levels.

We also analyzed a data set from a previous report which contained RNA-seq data from HAP1 cells in which KDAC8 was knocked out [50]. Analysis of these data resulted in identification of sets of up and downregulated genes compared to WT HAP1 cells (S8 Table). Although the cell types were not the same between this experiment and ours, we compared expression levels versus the matched wild-type data set for each experiment. Similar to KDAC6, comparison of the genes whose expression were affected by KDAC8 KO to those affected by catalytic inactivation revealed that these conditions led to distinct cellular consequences (Fig 4C). Loss of KDAC8 resulted in less than half the changes seen upon catalytic inactivation of KDAC8, and there was again only limited overlap between genes changed in each cell line. Despite the difference in the number of genes affected between the two cell lines, identification of GO terms associated with the gene expression changes in the HAP1 KDAC8 KO cell line produced a similar number of biological processes affected when compared to the HT1080 KDAC8H143A cell line (Fig 4D and S8 Table). Although about a quarter of the associated GO terms were shared between the two cell lines, with similarities involving processes related to transcription, RNA metabolism, and signaling, most affected processes were unique to one cell line or the other, again demonstrating that catalytic inactivation of KDAC8 was functionally distinct from KDAC8 KO.

**Fig 5:**
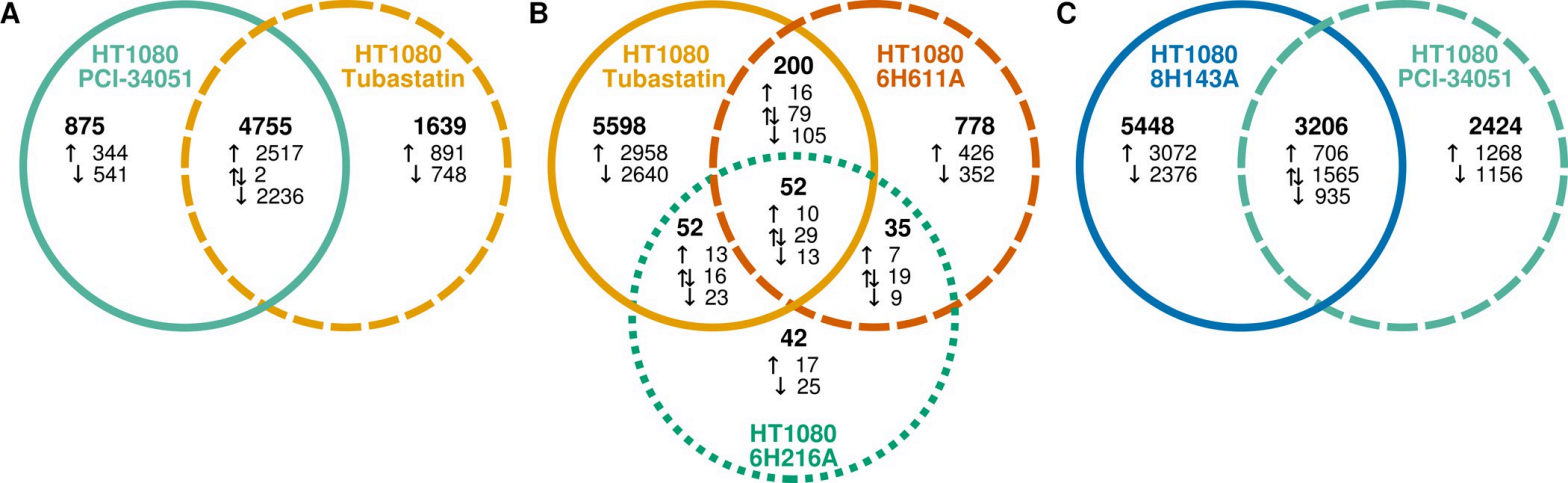
Effect of KDAC6 and KDAC8 catalytic inactivation on gene expression is not equivalent to treatment with inhibitors. (A) Venn diagram comparing the number of genes with significant changes in expression level for the two inhibitor treatments. (B) Venn diagram comparing the number of genes with significant changes in expression level for the Tubastatin A-treated cells to the KDAC6H216A and KDAC6H611A cell lines. (C) Venn diagram comparing the number of genes with significant changes in expression level for the PCI-34051-treated cells to the KDAC8H143A cell line. Gene counts are represented in the same way as for Fig 3.

We next evaluated the correlation between gene expression in our cell lines and the gene expression in cells treated with inhibitors previously reported to specifically target either KDAC6 (Tubastatin A) or KDAC8 (PCI-34051) [51, 52]. Wild-type HT1080 cells were treated with moderate doses of the inhibitors. We observed that the dosing treatment did not appear to slow cell growth as compared to untreated cells over the two days of treatment. Fig 5 summarizes the gene expression changes of the two inhibitors relative to each other and the catalytically inactive cell lines (also see S9 and S10 Tables). Unexpectedly, the two inhibitors have the majority of expression changes in common (Fig 5A). Moreover, the Tubastatin A treatment has only minor overlap with the HT1080 KDAC6H216A or HT1080 KDAC6H611A cell lines (Fig 5B). Although a small majority of genes with expression changes associated with PCI-34051 were also observed to change in HT1080 KDAC8H143A, nearly half of the overlapping set is “mixed”, indicating that PCI-34051 affected many of the same genes as HT1080 KDAC8H143A, but with the opposite effect (up-regulated instead of down-regulated or vice versa). Therefore, it is clear that it is not possible to recapitulate the cellular effects of our mutations with a simple inhibitor treatment. The GO terms associated with the two inhibitor treatments were also highly overlapping with each other, but only partially overlapping with the mutant cell lines (S5 and S6 Tables). To identify cellular functions being impacted by the inhibitor treatments but not by the inactivated KDACs, we used the set of genes appearing as only associated with the inhibitor in Fig 5B and 5C, and then analyzed the GO terms associated with the overlapping set of the two inhibitors (S6 and S7 Figs). Many cellular functions were found to be uniquely associated with inhibitor treatment (e.g., histone acetylation), while many other functions associated with both inhibitors overlapped with only one of the mutant cell lines (e.g., microtubule organization, DNA repair). No unique GO terms were associated with the set of genes specific to a single inhibitor (i.e., those with changed expression upon treatment with only one inhibitor and did not overlap with the mutant cell line(s) for the KDAC targeted by that inhibitor).

## Discussion

**Fig 6:**
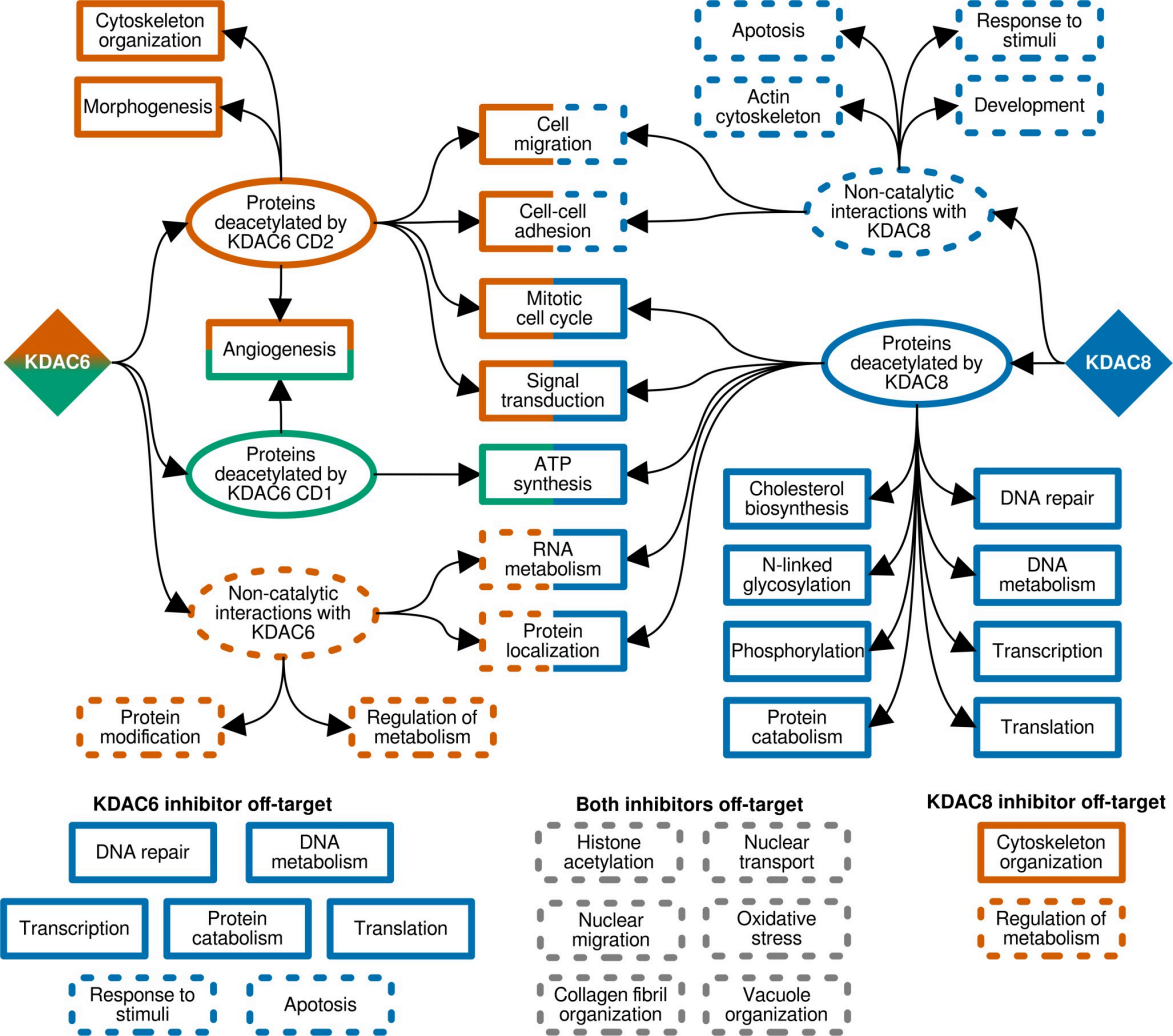
Schematic diagram of biological processes regulated by KDAC6 and KDAC8. Biological processes describing the major themes of significant GO terms associated with KDAC6 (CD1 green, CD2 red) and KDAC8 (blue) are shown. Solid boxes represent terms associated with deacetylation of proteins by the KDAC. Biological processes associated with loss of KDAC expression, but not associated with catalytic inactivation, are shown in dashed boxes. Off-target effects, which are defined as biological processes associated with the KDAC6 and KDAC8 inhibitors but not associated with the KDAC targeted by the inhibitor, are shown along the bottom. Processes common to both inhibitors, but not otherwise associated with either KDAC, are shown in dashed grey boxes. Off-target effects shown in colored boxes are only associated with KDACs not targeted by inhibitor.

In this report, we have focused on identifying the cellular consequences of inactivation of a single KDAC. We used the novel cell lines created in this work to identify key cellular processes regulated by deacetylation by particular KDACs. In the case of KDAC6, these functions could be further assigned to a specific catalytic domain of the enzyme. To synthesize the results from our experiments, we combined similar GO terms assigned to a particular cell line or treatment condition to describe the associated biological processes in the most straightforward manner. A graphical representation of this analysis highlights the major findings of our study and highlights several discrete conclusions (Fig 6), several of which are entirely consistent with previously reported experiments and which suggest impacts on some medically-relevant outcomes. As expected, deacetylation by KDAC6 CD1 regulates a smaller subset of processes than deacetylation by KDAC6 CD2. This result is consistent with prior work demonstrating that CD2 is responsible for most, but not all, of the catalytic activity of KDAC6 including tubulin deacetylation [10, 11]. KDAC6 CD2 deacetylation regulated more processes, including cytoskeletal organization, which was expected, as K40 of tubulin is a well-known substrate of KDAC6 CD2 which affects microtubule stability [53–55]. In addition, angiogenesis is a GO term associated with catalytic activity of both KDAC6 domains (Figs 3 and 6), and reduced angiogenesis has previously been attributed to KDAC6 inhibition and knockdown that is consistent with our observation [56, 57]. In fact, both CD1 and CD2 have been linked to angiogenesis through cortactin deacetylation [58]. Catalytic inactivation of KDAC8, in comparison, resulted in far more changes in gene expression than either of the KDAC6 mutations, and therefore, resulted in changes in many more biological processes spanning critical cellular functions. These results suggest that KDAC8 may target one or a small group of regulatory proteins that in turn control expression or regulation of a large number of downstream genes and/or proteins. It is also interesting that KDAC6 and KDAC8 share many functional themes, but for the majority of the themes, catalytic activity of only one of the KDACs is associated with the function (compare Fig 3C with Fig 6). A modality of overlapping function through distinct mechanisms, not necessarily modulated by catalysis, has not to our knowledge been previously proposed for these KDACs. A few themes are associated with catalytic activity of both KDAC6 and KDAC8, and may represent overlapping specificity in substrates or alternative targets to regulate the same outcomes.

While there are only a handful of well-characterized substrates for KDAC6 and KDAC8, most of them are known to participate in biological processes that we have identified in this study [9]. For example, the KDAC6 substrate cortactin, in addition to its proposed role in angiogenesis, is involved in cytoskeletal organization, morphogenesis, and signal transduction, which have all been identified in this study as processes affected by KDAC6 CD2 [58–60]. Likewise, ARID1A, a substrate of KDAC8, is a well-studied transcriptional regulator which is known to impact several processes linked to KDAC8 in this study, namely cell cycle, DNA/RNA metabolism, DNA repair, and transcription [20, 61]. Thus, we hypothesize that regulation of key proteins through acetylation/deacetylation can have major impacts on cellular function. This explains how misregulation of a particular KDAC can lead to multiple phenotypes and disease states. Several essential processes, such as cell cycle and signal transduction, were affected by both KDAC6 and KDAC8 deacetylation. It is possible that under certain circumstances KDAC6 and KDAC8 (or other KDACs) can perform overlapping functions, and compensate for one another if necessary. Alternatively, they may affect expression of the same genes and cellular processes by deacetylating unique targets that function together to regulate a biological process. The cell lines will be useful in future studies for identifying direct substrates of KDAC6 and KDAC8, using either a targeted approach to confirm putatively identified substrates from cell-based or *in vitro* studies or using a mass-spectrometry based proteomics approach that will allow for more directly linking the identified cellular processes to specific changes in acetylation. Understanding how and when KDACs work together to regulate a single cellular outcome is also an interesting avenue for future study.

Producing cell lines in which a KDAC was genetically inactivated, but which retained endogenous expression, allowed for comparison to cells with complete loss of KDAC expression. There are few precedent reports that systematically compared the consequences of these modes of perturbation for the same protein. While it is frequently assumed that enzyme inhibition (in principle, roughly analogous to our catalytically inactivated enzymes) and gene knockout/knockdown will produce very similar results in cells, it has been established that there are instances, such as in the kinase field, where inhibition and knockdown of a particular enzyme do not produce the same phenotype for biologically important reasons [62]. In addition to the processes regulated by KDAC6 and KDAC8 deacetylation identified in genetically inactivated lines, we also identified a smaller set of processes that were only perturbed when KDAC6 and KDAC8 were not present (i.e., knockdown or knockout). These represent the effects of non-catalytic functions of the enzymes. While we expected some processes to fall into this category, we were surprised by the number of genes and processes that were unique to catalytic inactivation, meaning they were perturbed when the KDAC was present but inactive; however, the genes regulating the process were expressed normally when the KDAC was not present at all (Fig 4). This represents an interesting set of cellular effects that would not have been observed using only standard techniques to study protein function by modulating protein levels in cells.

There is some evidence in the literature that knockout and knockdown experiments are not equivalent, as gene knockouts allow for compensatory mechanisms to occur in cells, while knockdown shows the acute state of cells after losing a protein [63]. Thus, different experimental approaches measure distinct acute and chronic effects on cells: (1) traditional short-term inhibitor treatment is acute inactivation of catalysis while retaining non-catalytic functions; (2) knockdown and degradation-based inhibitor methods are acute inactivation of both catalytic and non-catalytic functions; and (3) knockout is chronic loss of both catalytic and non-catalytic functions. In contrast, the genetic inactivation of KDACs used here demonstrates a fourth approach: the chronic effects of losing only catalytic activity, which may better represent the effects of a prolonged treatment strategy compared to the other experimental methods. Initially, we hypothesized that changes resulting from catalytic inactivation would represent a subset of the total changes observed upon knockout or knockdown of a particular KDAC. Comparison of our data sets clearly demonstrate that this was not the case. We observed similar overlap with the HAP1 KDAC8 knockout cell line versus our HT1080 8H143A cell line and with the HT1080 KDAC6 knockdown line compared to either of our two genetically inactivated lines, even though the KDAC8 comparison was not in the same cell line. Changes caused by genetic inactivation of KDACs were distinct and not highly overlapping with the effects of losing the entire protein (Fig 4). This may be in part attributed to gain-of-function effects associated with even endogenous level expression of catalytically-inactive KDACs. Our results add to a growing body of literature that suggests that the manner in which enzymes are studied in cells greatly affects the experimental outcomes, underscoring the importance of utilizing a proper experimental tool to answer particular biological questions.

Furthermore, we observed that chemical inhibition of KDAC6 and KDAC8 results in effects that are also distinct from our genetic inactivation of the KDACs (Figs 5 and 6 and S4 and S5 Tables). These data represent off-target effects of the inhibitors. Based on IC_50_ values, Tubastatin A is considered selective for KDAC6; however, IC_50_ values can also be measured for KDAC8 (57x higher than KDAC6) and KDAC1 (∼1000x higher than KDAC6) [51]. Similarly, PCI-34051, known as a KDAC8-specific inhibitor, can also inhibit KDAC6 and KDAC1, albeit with approximately 300-fold and 400-fold less specificity based on IC_50_ values, respectively [52]. Based on these observations, it is certainly possible, depending on experimental conditions, that either of these inhibitors could at least partially inhibit more than one KDAC in cells, leading to off-target effects. Off-target effects have previously been reported for Tubstatin A at concentrations 10-fold greater than we utilized, but not at the 1 μM concentration used in our study [64–66]. It is interesting to note that although KDAC6 and KDAC8 belong to different classes of deacetylases, they show the most similar specificity profiles for both of the inhibitors used in this work. Moreover, the appearance of histone acetylation as a major GO term upon inhibitor treatment (Fig 6), as well as other epigenetic-related terms absent from the mutant cell lines, strongly suggests that the inhibitors are binding to a common target other than KDAC6 or KDAC8. Recently, MBLAC2 was identified as a protein, not closely related to the KDACs, that is frequently inhibited by KDAC inhibitors [67]. Such effects, as well as binding to KDACs other than the intended target, are likely the cause of the observed differences in gene expression. Certainly, this issue could limit the interpretation of experiments aimed at identifying targets and cellular functions of KDACs from inhibitor studies. Furthermore, even small off-target effects could affect the success of KDAC inhibitors as therapeutics, and having a tool to understand when and if this is occurring for given inhibitors is valuable for pre-clinical drug studies. Our genetically engineered cell lines provide an alternate system for identifying the consequences of shutting off a single KDAC, and potentially for understanding how well various inhibitors mimic complete, specific inactivation of a single KDAC. Future work will evaluate the extent to which gene expression differences are a result of the time scale of inhibitor treatment versus the chronic state of inactivation in the cell lines, as well as the effects on gene expression in the mutant cell lines following inhibitor treatment.

While the CRISPR/Cas9 system has been extensively used to engineer cell lines, the technique is commonly used to efficiently knockout particular genes of interest by inserting or deleting bases at a specific genomic location. Here, we have instead inactivated the enzyme with a missense mutation in human cells, while retaining expression of the mutant gene from the endogenous locus. Importantly, the gene variants replace the wild-type genes under control of the native promoter and without adding additional sequences, such as a tag. Theoretically, this approach circumvents limitations associated with other approaches, such as changes in expression and off-target effects of chemical inhibitors, although with trade-offs in greater complexity of execution and longer time scales that may allow for some unexpected adaptive behaviors. Indeed, we observed that our cell lines behaved differently to cell lines where the same KDACs were manipulated by these more traditional methods. Taken together, demonstration of these differences unequivocally demonstrated that investigating the catalytic functions of particular KDACs using these cell lines is not equivalent to doing so using other, more common methodologies. We hypothesize that these cells lines will provide additional information about the consequences of loss of activity of a single KDAC, which will be useful for understanding KDAC function and therapeutic possibilities. This design strategy can also easily be applied to other KDACs as well as other classes of enzymes, as the advantages for the enzymes studied here should be broadly applicable to other enzymes.

## Supporting information

S1 Raw images Raw uncropped immunoblots. (PDF)

S1 Fig GO graph for HT1080 KDAC6H216A based on down-regulated genes. (PDF)

S2 Fig GO graph for HT1080 KDAC6H611A based on up-regulated genes. (PDF)

S3 Fig GO graph for HT1080 KDAC6H611A based on down-regulated genes. (PDF)

S4 Fig GO graph for HT1080 KDAC8H143A based on up-regulated genes. (PDF)

S5 Fig GO graph for HT1080 KDAC8H143A based on down-regulated genes. (PDF)

S6 Fig GO graph for overlapping gene expression changes of wild-type HT1080 treated with Tubastatin A or treated with PCI-34051 and which do not overlap with the target KDAC for each inhibitor, based on up-regulated genes. (PDF)

S7 Fig GO graph for overlapping gene expression changes of wild-type HT1080 treated with Tubastatin A or treated with PCI-34051 and which do not overlap with the target KDAC for each inhibitor, based on down-regulated genes. (PDF)

S1 Table Calculated ΔΔC_T_ values for KDAC6 and KDAC8 in each cell line. (PDF)

S2 Table Significant gene expression changes in HT1080 KDAC6H216A relative to wild-type. (XLSX)

S3 Table Significant gene expression changes in HT1080 KDAC6H611A relative to wild-type. (XLSX)

S4 Table Significant gene expression changes in HT1080 KDAC8H143A relative to wild-type. (XLSX)

S5 Table Significant GO terms identified by the less stringent method for all cell lines and treatments. (XLSX)

S6 Table Significant GO terms identified by the more stringent method for all cell lines and treatments. (XLSX)

S7 Table Significant gene expression changes in HT1080 KDAC6KD relative to wild-type. (XLSX)

S8 Table Significant gene expression changes in HAP1 KDAC8KO relative to wild-type. (XLSX)

S9 Table Significant gene expression changes in wild-type HT1080 treated with Tubastatin A relative to untreated. (XLSX)

S10 Table Significant gene expression changes in wild-type HT1080 treated with PCI-34051 relative to untreated. (XLSX)
