# Supplementary material for: Endogenous expression of inactive lysine deacetylases reveals deacetylation-dependent cellular mechanisms
Source: PLoS One. 2023 Sep 18;18(9):e0291779. doi: 10.1371/journal.pone.0291779 (PMC10506724; doi:10.1371/journal.pone.0291779)
Supplement: S1 Raw images — (PDF) [file pone.0291779.s001.pdf]

S1 Fig. Raw uncropped immunoblots

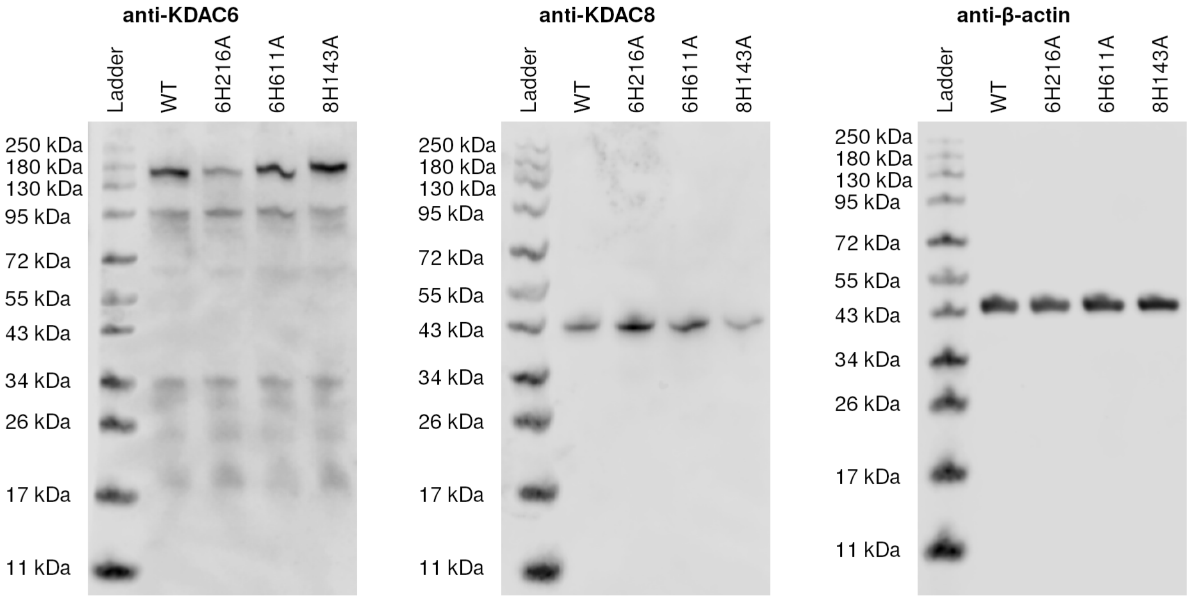

KDAC expression Fig 2B. The expected masses are 131 kDa (KDAC6), 42 kDa (KDAC8), 42 kDa (β-actin). Some secondary bands in the KDAC6 blot correspond to masses of reported isoforms. Images captured and prepared as described in the Materials and Methods.

S1 Fig. Raw uncropped immunoblots (continued)

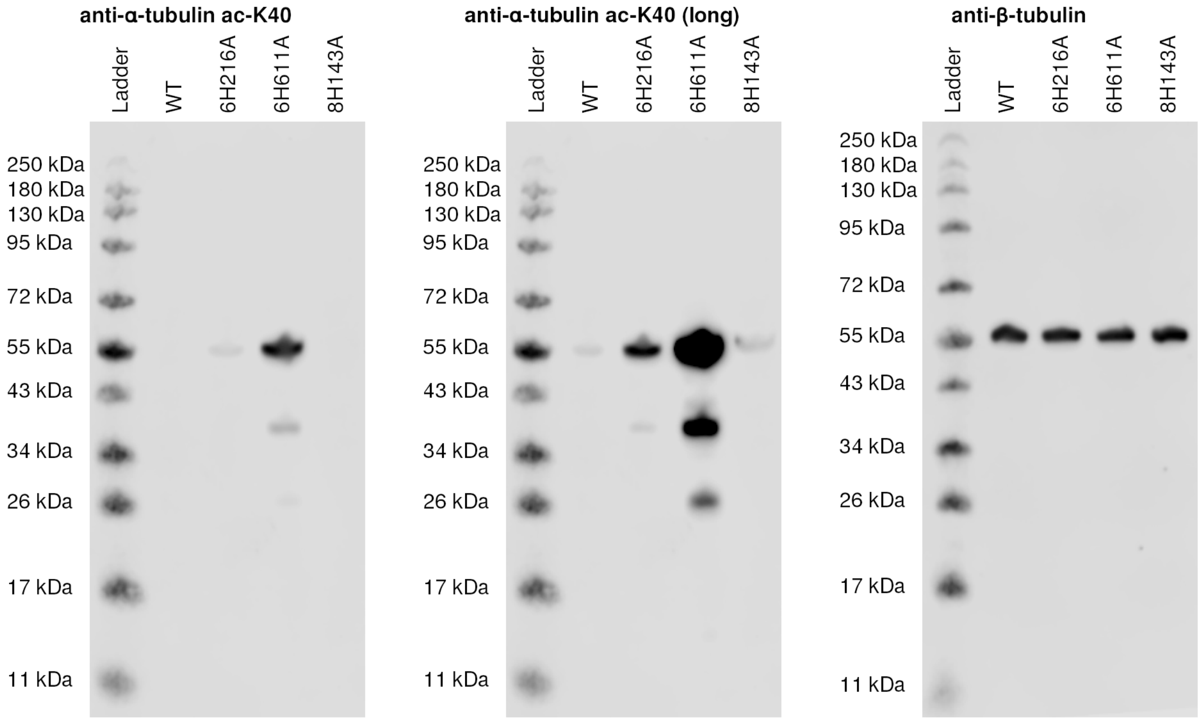

Tubulin acetylation Fig 2C. The expected mass of α/β-tubulin is 50 kDa but is observed to run slightly larger compared to the ladder bands. Secondary bands correspond to masses of α-tubulin isoforms. Because α-tubulin and β-tubulin form obligate heterodimers and are transcriptionally linked, α-tubulin and β-tubulin levels are tightly coupled. Images captured and prepared as described in the Materials and Methods.

S1 Fig. Raw uncropped immunoblots (continued)

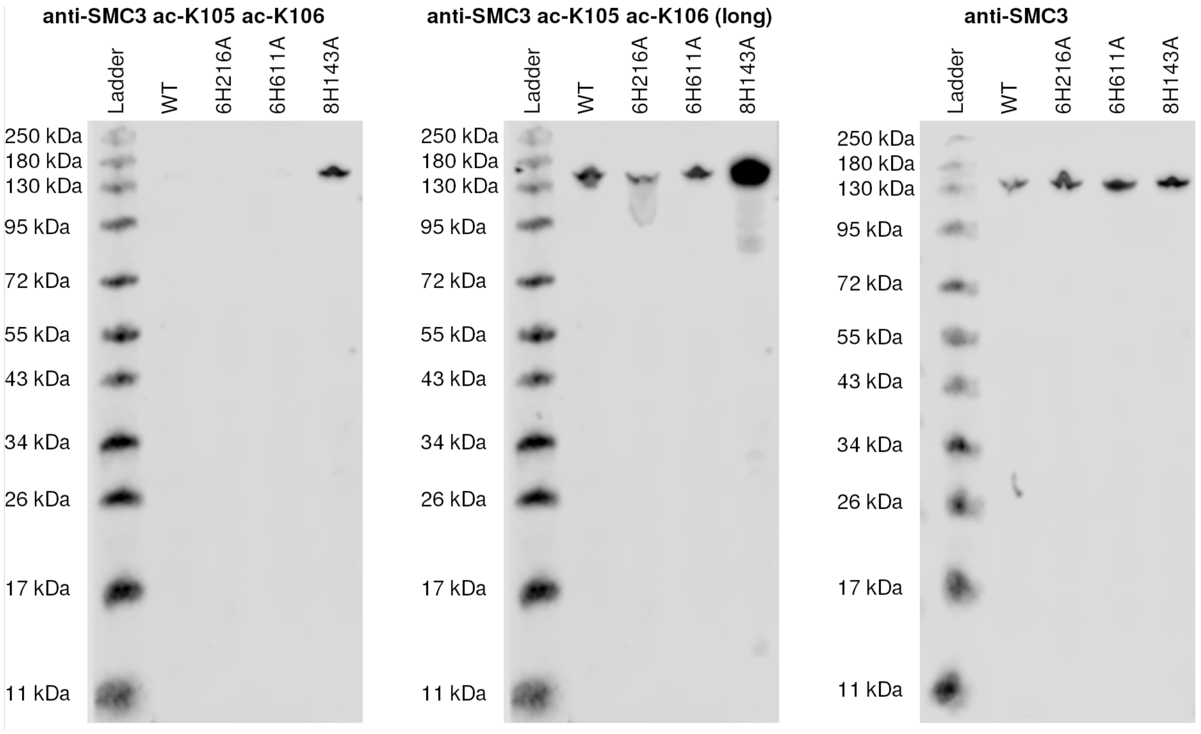

SMC3 acetylation Fig 2D. The expected mass of SMC3 is 142 kDa. Images captured and prepared as described in the Materials and Methods.

S1 Fig. Raw uncropped immunoblots (continued)

anti-histone H3 ac-K9

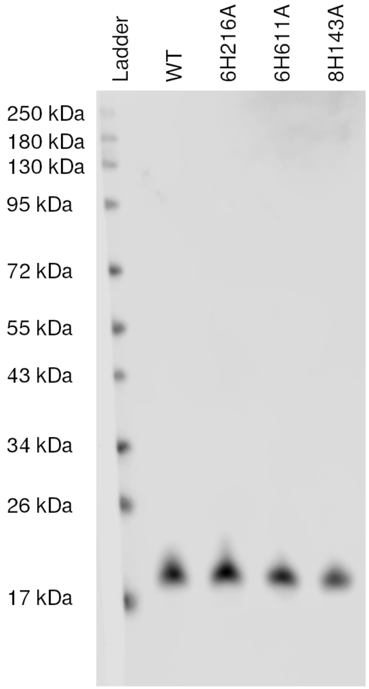

anti-histone H3 ac-K18

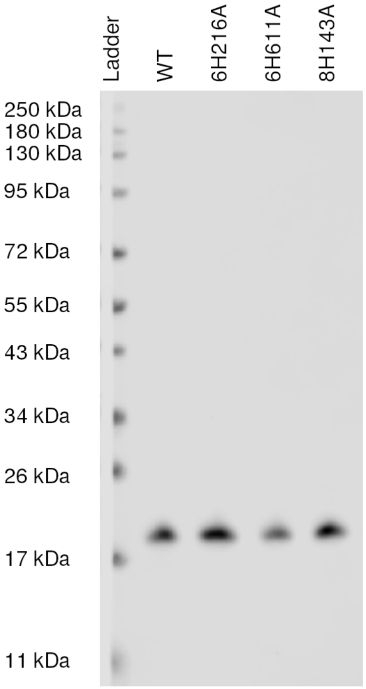

anti-histone H3 K27

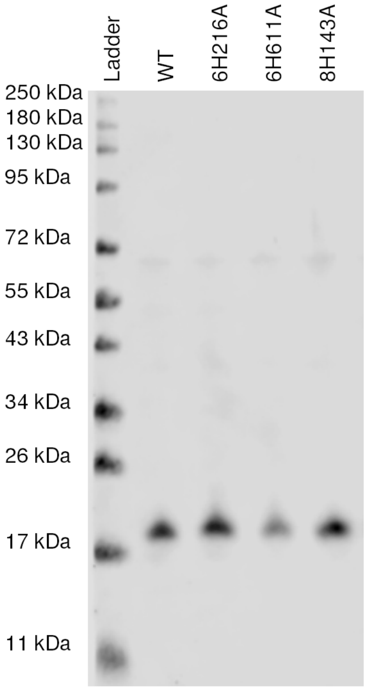

anti-histone H3

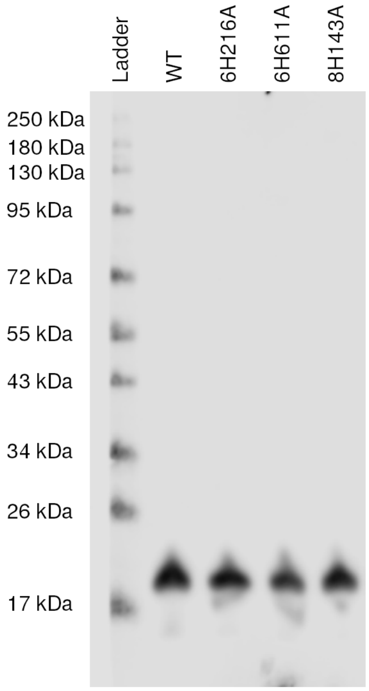

Histone H3 acetylation Fig 2E. The expected mass of histone H3 is 15 kDa, but it is observed to run slightly larger than expected compared to ladder bands. Images captured and prepared as described in the Materials and Methods.
