# Supplementary material for: Endogenous expression of inactive lysine deacetylases reveals deacetylation-dependent cellular mechanisms
Source: PLoS One. 2023 Sep 18;18(9):e0291779. doi: 10.1371/journal.pone.0291779 (PMC10506724; doi:10.1371/journal.pone.0291779)
Supplement: S1 Table — (PDF) [file pone.0291779.s009.pdf]

S1 Table. Calculated $\Delta\Delta C_T$ values for KDAC6 and KDAC8 in each cell line.

KDAC6			KDAC8		
**6H216A**	**6H611A**	**8H143A**	**6H216A**	**6H611A**	**8H143A**
0.50	0.62	0.78	1.42	1.30	1.47
0.58	0.77	0.78	1.40	1.37	1.43
0.62	0.66	0.79	1.51	1.35	1.74
0.59	0.75	0.71	0.82	1.06	1.34
0.61	0.74	0.72	0.78	0.93	1.49
0.62	0.79	0.59	0.96	1.03	1.30
0.72	1.24		0.94	0.95	1.28
0.75	1.06		0.92	0.89	1.32
0.54	1.13				1.38
					0.97
					0.79
					0.93
					0.76
					0.72
