# Supplementary material for: Endogenous expression of inactive lysine deacetylases reveals deacetylation-dependent cellular mechanisms
Source: PLoS One. 2023 Sep 18;18(9):e0291779. doi: 10.1371/journal.pone.0291779 (PMC10506724; doi:10.1371/journal.pone.0291779)
Supplement: S1 Fig — (PDF) [file pone.0291779.s002.pdf]

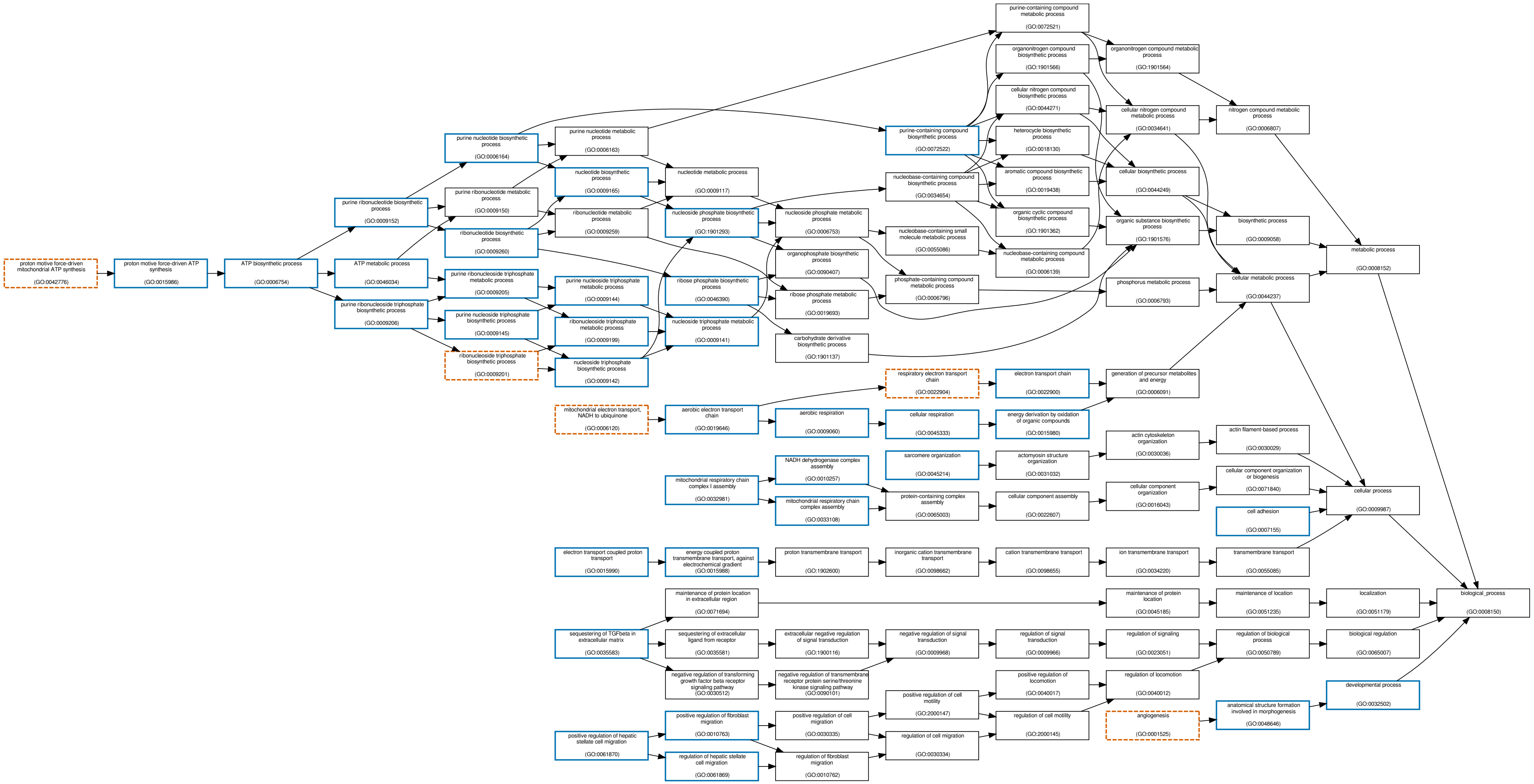

S2 Fig. GO graph for HT1080 KDAC6H216A based on down-regulated genes. Red dashed boxes are terms that were significant by the stringent method (and therefore also the non-stringent method). Blue solid boxes are terms that were significant only by the less stringent method. Black boxes are terms that were not significant, but which exist along the shortest path between a significant term and biological_process. Lines represent *is_a* and *part_of* relationships. This figure is text-searchable.
